# Moderating Effect of Mindfulness on the Influence of Stress on Depression According to the Level of Stress among University Students in South Korea

**DOI:** 10.3390/ijerph17186634

**Published:** 2020-09-11

**Authors:** Kwang-Hi Park, Hyunlye Kim, Jaehee Kim

**Affiliations:** 1 College of Nursing, Gachon University, Incheon 21936, Korea; parkkh@gachon.ac.kr; 2 Department of Nursing, College of Medicine, Chosun University, Gwangju 61452, Korea; 3 Department of Nursing, College of Science and Technology, Daejin University, Pocheon 11159, Korea; hjw9266@daejin.ac.kr

**Keywords:** stress, mindfulness, depression, university students

## Abstract

Stress and depression are representative of the mental health problems of university students worldwide. This cross-sectional study explored the moderating effect of mindfulness on the influence of stress on depression according to the degree of life stress. The participants were 738 university students in years 2–4 in five 4-year universities in South Korea. Depression was positively correlated with stress and negatively with mindfulness at a statistically significant level. In multiple regression analysis, stress was found to have an effect by increasing depression, and mindfulness by relieving depression. In the moderated multiple regression analysis, mindfulness had a moderating effect on the impact of stress on depression only in low-stress groups, showing that the interaction of stress with mindfulness was significantly negative (β = −0.11, t = −2.52, *p* = 0.012) and the inclusion of this interaction significantly increased the explanatory power for depression variation (F change 6.36, *p* = 0.012) in the full model. In conclusion, we suggest considering stress levels in the development of mindfulness-based intervention strategies to effectively manage the depression of university students.

## 1. Introduction

University students experience a variety of stresses due to problems related to their employment, academic achievement, economic status, values, and interpersonal relationships, as they undergo a transitional developmental stage from late adolescence to early adulthood. According to the 2018 Korea National Health and Nutrition Examination Survey released by the Ministry of Health and Welfare, the stress perception rate (the percentage of people who feel “severe” or “a lot” of stress in their daily life) of all adults over the age of 19 was 27.3%, and for ages 19–29 years including university students, it was 35.7%, the highest compared to all other age groups [1]. Studies on the mental health of Korean university students show that the stress of university students is increasing and, as a result, various psychological problems such as depression, anxiety, substance abuse, and behavioral addictions have also increased [2,3]. In particular, Korean university students enter university through fiercely competitive entrance exams, and they are highly likely to experience extreme life stress due to various social demands as they face the constant academic burden and the uneasy reality of their career and future after graduation [3].

Depression is one of the most prevalent mental health problems among university students and is typically marked by sad feelings or negative emotions toward oneself [4,5]. Depression has high associations with stress and suicide potential, and reports emphasize that efforts to manage university students’ stress and reduce depression are urgent [2,6]. The various negative effects of stress depend on the individual’s internal and external resources and environment [7]. Some of the known variables involved with stress and psychological difficulties include mindfulness, ego resilience, optimism, intolerance of uncertainty, cognitive emotion regulation, coping style, problem solving ability, spiritual meaning, and social support [3,8,9,10,11,12,13].

This study focused on mindfulness, which is widely recognized medically, in psychological counseling, and social welfare as a moderator for stress outcomes. Mindfulness is related to the particular qualities of attention and awareness that can be traditionally developed through meditation; it is defined as “the awareness that emerges through paying attention on purpose, in the present moment, and nonjudgmentally to the unfolding of experience moment by moment” [14]. To cultivate mindfulness, both informal methods such as mindfully walking and showering and formal methods such as meditation programs can be used [15]. Furthermore, mindfulness is greatly enhanced through regular disciplined practice on a daily basis [16]. Mindfulness is the basic concept of a strength-focused strategy that has been in the spotlight in the mental health setting, and evidence has accumulated on the positive effects of mindfulness-based interventions [17]. For example, since Dr. Jon Kabat-Zinn developed a program (Mindfulness-Based Stress Reduction, MBSR) to provide a coping resource through mindfulness for patients suffering from psychological illnesses in 1979 [16], it has been reported to have a positive effect on stress in patients with a variety of physical and psychological problems [18,19,20,21]. Mindfulness-based strategies also contribute to lowering stress and depression in university students [22,23,24,25,26].

Russell and Siegmund [17] emphasized understanding the subject’s characteristics and context to maintain the suitability of a mindfulness-based strategy and enhance its effectiveness. Several researchers have conducted studies to investigate the mediating or moderating effects of mindfulness on the relationship between stress and depression among nurses, nursing students, and undergraduate students, but the results have not been consistent [13,27,28]. In Park’s study of Korean undergraduate students, mindfulness showed no significant moderating effect on the relationship between stress and depression [13]. However, the mediating effect of mindfulness in Korean nursing students [28] and its moderating effect in Chinese intensive care unit nurses [27] were significant. A mediating effect assumes, and also verifies, the mechanism of the relationship between variables, whereas the moderating effect analysis determines whether a variable affects the strength or direction of the relationship between the other two variables [29,30]. Jimenez et al. [31] emphasized the need to understand the regulatory mechanism of mindfulness by presenting a conceptual model of mindfulness and its influence on depression. Ramli et al. [32] suggested that mindfulness may work differently depending on the level of stress by showing that university students exhibited a low degree of mindfulness when facing high stress. In this study, we aimed to investigate the role of mindfulness as a moderator in the relationship between stress and depression according to the level of stress among Korean university students. The results of this study will provide basic data for devising more effective mindfulness intervention strategies according to the level of stress.

## 2. Materials and Methods

### 2.1. Study Design and Participants

**Figure 1:**
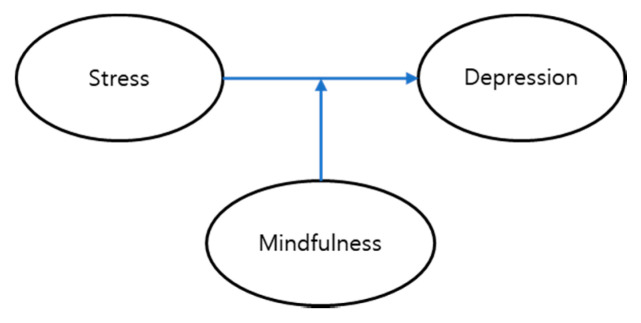
A hypothetical model of the moderating effect of mindfulness.

This cross-sectional study was conducted to explore the moderating effect of mindfulness on the relationship between stress and depression according to the level of stress in university students (Figure 1). The participants were 738 university students in years 2–4 at five 4-year universities in metropolitan and provincial areas of South Korea.

### 2.2. Data Collection

We used a non-probability convenient extraction sampling method. The survey was conducted from May to June 2019 at two universities in the metropolitan area and three local universities. The researcher explained the purpose and method of the research to the head of the department in the relevant university in advance and obtained permission for data collection. After receiving an explanation of the content and method of the study, the university students agreed in writing to participate in the survey and then responded to the self-reported questionnaire. Of the 750 questionnaires collected, 738 were used for the analysis after excluding questionnaires containing missing values or unreliable responses.

### 2.3. Ethical Considerations

This study was approved by the institutional review board of a university (approval no. 1044396-201904-HR-069-01; date. 2019. 4. 30). After being informed of the purpose and content of this study, and also with regard to how to complete the questionnaire, their ethical rights as a research participant, the confidentiality and anonymization of data, and confirmation of data not being used for purposes other than research, potential participants voluntarily signed a written consent form.

### 2.4. Instruments

#### 2.4.1. Life Stress

To measure their life stress, the Revised Life Stress Scale for college students was used, which was developed by Chon et al. [33]. Each item was designed to respond to the frequency and importance of the experience on a 4-point Likert scale from “Not at all (0)” to “Frequently (3)”. This tool consists of 50 items, and the total score range is 0–150 points; the higher the score, the higher the stress. It consists of eight life stress areas (interpersonal relationship with friend, lover, family, and faculty; task-related stress regarding grades; economic stress; stress about the future; and stress related to values). The internal reliability (Cronbach’s alpha) of this tool was 0.90 in a study by Lee [11] and 0.93 in this study.

#### 2.4.2. Mindfulness

Mindfulness was measured using the Korean version of the Cognitive and Affective Mindfulness Scale-Revised (CAMS-R), validated by Cho [34]; the CAMS-R was developed by Feldman et al. [35]. This tool is a 4-point Likert scale from “Rarely (1)” to “Almost always (4)” with 10 questions. The total score range is 10–40 points; the higher the score, the higher the level of mindfulness. It also contains sub-factors of awareness (four items), attention (four items), and acceptance (two items). The Cronbach’s alpha value was 0.70 in the study of Cho [34] and 0.80 in this study.

#### 2.4.3. Depression

Depression was measured using an integrated Korean version of the Center for Epidemiologic Studies Depression Scale (CES-D) developed by Chon et al. [36] based on the original by Radloff [37]. Each item was designed to respond to the frequency of depression experienced in the past week on a 4-point Likert scale from “Very rarely (less than 1 day; 0)” to “Almost every day (5–7 days; 3)”. This tool consists of 20 items, and the total score range is 0–60 points; the higher the score, the greater the depression. The Cronbach’s alpha value was 0.91 in the study by Chon et al. [36] and 0.91 in this study.

### 2.5. Data Analyses

To analyze the results of this study, SPSS Statistics for Windows, Version 22.0. (IBM Corp. Armonk, NY, USA) was used for the collected data. Descriptive statistics were performed on demographic characteristics and main variables. ANOVA was performed to compare the differences in stress, mindfulness, and depression according to the general characteristics. The associations between stress, mindfulness, and depression were explored using Pearson’s correlation analysis. To identify the role of mindfulness as a moderator, a moderated multiple regression using hierarchical regression analysis including interaction term input was performed. General characteristics were set as a control variable, stress as an independent variable, mindfulness as a moderating variable, and depression as a dependent variable. The existence of the moderating effect was judged by confirming the probability of significance of the interaction term, the amount of R^2^ and F changes in the final full model [38,39].

## 3. Results

### 3.1. Descriptive Statistics

The average age of the participants was 21.66 ± 1.88 and ranged from 19 to 31 years, with 61.0% in the age group 20–22 years. There were more women (74.4%) than men. University students’ majors were mainly in nursing (39.4%), business administration (35.5%), and tourism (19.8%). In terms of satisfaction with university life, respondents answered “satisfied” (40.9%), “moderate” (47.4%), or “unsatisfied” (11.7%). With regard to the major variables in this study, the average stress score was 34.00 ± 18.21 points (0.68 ± 0.36 of 3 possible points per item) and ranged from 3 to 90. The average mindfulness score was 24.72 ± 4.89 (2.47 ± 0.49 of 4 possible points per item) and ranged from 13 to 40. The average depression score was 18.13 ± 10.19 (0.91 ± 0.51 of 3 possible points per item) and ranged from 0 to 56.

### 3.2. Differences in Stress, Mindfulness, and Depression by General Characteristics

Table 1 shows the differences in stress, mindfulness, and depression according to general characteristics. Stress showed statistically significant differences according to sex (t = −6.27, *p* < 0.001), age group (F = 7.15, *p* = 0.001), year level (F = 6.81, *p* = 0.001), subjective economic status (F = 42.35, *p* < 0.001), and subjective health status (F = 42.44, *p* < 0.001). Specifically, the stress level was higher in female than in male students, in the 20–22-year age group than in older students, in 3rd year students than in 2nd year students, and when subjective economic status and subjective health status were worse. Mindfulness showed statistically significant differences according to subjective economic status (F = 14.95, *p* < 0.001) and subjective health status (F = 22.95, *p* < 0.001). Specifically, the level of mindfulness was higher when subjective economic status was positive and when subjective health status was better. Depression showed statistically significant differences according to sex (t = −4.84, *p* < 0.001), age group (F = 4.38, *p* = 0.013), subjective economic status (F = 17.17, *p* < 0.001), and subjective health status (F = 78.05, *p* < 0.001). Specifically, the level of depression was higher in female than in male students, in the 20–22-year age group than in the 23-or-older age group, when the subjective economic status was poor or moderate, and when the subjective health status was worse. There were no differences according to university students’ majors and university life satisfaction.

### 3.3. Correlations between Stress, Mindfulness, and Depression

Correlations among key variables are presented in Table 2. Stress was negatively correlated with mindfulness (r = −0.284, *p* < 0.001). Depression was positively correlated with stress with a steep slope (r = 0.625, *p* < 0.001) and negatively correlated with mindfulness (r = −0.431, *p* < 0.001). All three variables showed highly significant correlations.

### 3.4. Moderating Effect of Mindfulness According to the Degree of Stress

Statistical analyses to confirm the moderating effect were conducted in three groups: the total group, the high-stress group, and the low-stress group. The low- and high-stress groups were composed of 377 and 361 students, respectively, based on the median value of 31. Descriptive statistics for the low- and high-stress groups are presented in Table 3. The categorical variable among the independent variables was analyzed by determining the reference group (“male” in sex; “~19” in age; “good” in subjective economic status; “satisfied” in subjective health status), and treating them as dummy variables. In the previous multiple regression analyses, stress (+) and mindfulness (−) were predictors affecting depression in the total group (stress β = 0.50, t = 16.47, *p* < 0.001; mindfulness β = −0.25, t = −8.89, *p* < 0.001), the high-stress group (stress β = 0.31, t = 6.49, *p* < 0.001; mindfulness β = −0.25, t = −5.54, *p* < 0.001), and the low-stress group (stress β = 0.32, t = 6.78, *p* < 0.001; mindfulness β = −0.33, t = −7.16, *p* < 0.001) and showed explanatory powers (adjusted R^2^) of 0.495, 0.299, and 0.347, respectively.

In the moderated multiple regression analyses, there was no moderating effect in the total group (β = −0.03, t = −0.22, *p* = 0.828) and the high-stress group (β = 0.28, t = 1.04, *p* = 0.298). Table 4 shows the results of the moderated multiple regression analysis in the low-stress group. This regression model’s Durbin–Watson value was 2.01 (around 2), which confirmed no autocorrelation problem was detected. The possibility of multicollinearity that can occur in a full model with an interaction term is minimized by centering the two predictors (stress, mindfulness) around the mean [40]. After this process, the variance inflation factor (VIF) value of the independent variables in the final model 4 was 1.07~2.80 (<10), and there was no problem of multicollinearity. As reported in Model 4, the interaction of stress with mindfulness was significantly negative (β = −0.11, t = −2.52, *p* = 0.012) and the inclusion of this interaction significantly increases the explanatory power for depression variation (F change 6.36, *p* = 0.012). This suggests that mindfulness had a moderating effect on depression through its interaction with stress.

**Figure 2:**
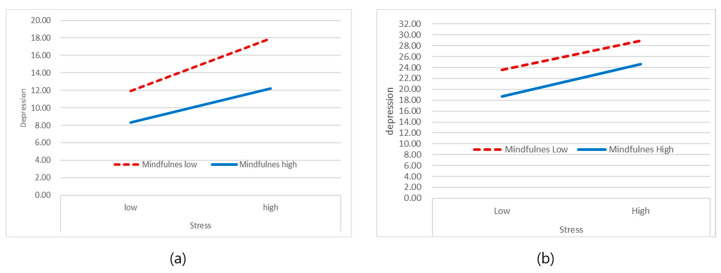
Moderating effect of mindfulness in the low- (**a**) and high- (**b**) stress groups.

The plot of the slope for the low- and high-stress group is shown in Figure 2. In plotting simple slopes in the low-stress group, the change in depression with increased stress in the group with low mindfulness was greater, showing a steeper slope than that in the group with high mindfulness (Figure 2). It indicated that the mitigating effect of mindfulness on depression associated with stress was more pronounced by showing a gentler slope. In other words, mindfulness plays a role as a moderator by alleviating the influence of stress on depression in the low-stress group. On the other hand, in the high-stress group, little difference is seen in the slope of the group’s depression change according to the level of mindfulness.

## 4. Discussion

Stress and depression are key mental health problems for university students around the world, leading to considerable research. We suggest the implications from the results of this study indicate that the consideration of stress, depression, and their predictive factors should be studied in university students in various countries. In this study, university students’ life stress (0.68 ± 0.36) and depression (0.91 ± 0.51) scores were lower than the median values on the measures’ 0–3-point scales. Compared with results from other studies using the same tool, these scores were lower than those for stress (mean 39.50; *N* = 120) [28] and depression (mean 19.90; *N* = 283) [41] in nursing students in Korea. Furthermore, stress and depression were found to be significantly higher in female students than in male students, consistent with the results of previous studies [5,14,42,43]. Studies conducted in Korea [43] and the United States [44] emphasized the need for a sex-specific approach by suggesting that different factors affect stress, depression, and their relationships in male and female university students. Several researchers in India [4] and the United States [45] conducted mental health studies centered on female university students, focusing on these sex differences. Considering gender issues will contribute to devising effective ways to address the mental health problems of university students.

In this study, stress and depression were highest in the group aged 20–22 years and in the third year. These results are thought to be due to the increasing amount of study and worry about the future (particularly employment acquisition) in the 3rd year. However, in studies conducted in Turkish university students [42] and Hong Kong nursing students [46], the stress levels were highest among lower-year university students, showing contradictory results. In a study of nursing students in Korea, the depression scores of second-year students were significantly the highest [47]. This seems to be because the social context surrounding university students in each country is different. In our study, stress and depression were also at the highest statistically significant levels when subjective economic and health conditions were unsatisfactory. These results are generally consistent with those of previous studies targeting college or university students [5,46,47]. The socioeconomic level, physical health status, and psychosocial maladjustment results display complex and bidirectional relationships that need to be explored in greater depth in the future. In addition, stress and depression are closely related to each other. In this study, these two variables were positively correlated, and stress was a significant predictor of depression, consistent with the results of many previous studies in India [4], Korea [3,5,12,43,48,49,50], and the United States [51]. Therefore, it would be effective to deal with these two mental health problems by considering them together.

Mindfulness has been noted as an individual’s strengthening element related to mental health outcomes. In this study, mindfulness was found to be significantly highest when subjective economic status and health status were satisfactory. These findings suggest that mindfulness can be most functionally exerted when the inner state and the environmental state are positive. Furthermore, in this study, mindfulness was negatively correlated with stress and depression and was a significant negative predictor of depression. Consistent results were found in many previous studies. Mindfulness was negatively correlated with stress in studies of nursing students in Korea [52] and the United States [53]. In a study of university students in Malaysia, mindfulness was reported to be negatively correlated with stress; it reduced stress and acted as a mediator between negative mental health outcomes [32]. A study that narratively reviewed 57 studies on the effects of mindfulness meditation on stress and anxiety, concluded that mindfulness training has a promising effect on reducing stress and anxiety in university students [54]. Mindfulness was found to have a negative correlation with depression in studies of university students in Korea [52,55], and the United States [31,44], consistent with our findings. As demonstrated in the mindfulness-based intervention studies in Spanish university students [22] and in Thai nursing students [56], the use of mindfulness in managing stress and depression in university students is expected to be an effective strategy.

In this study, mindfulness was found to have a moderating effect that reduces the effects of stress on depression in low-stress groups. This was consistent with the results of a study in Australian law students [57] and in Chinese intensive care nurses [27] that used moderated multiple regression analysis to verify that mindfulness acts as a protective factor in alleviating the negative effects of stress on depression. However, in our study, mindfulness did not buffer the effects of stress on depression when the stress level was high. A study of Malaysian university students showed that students experiencing academic stress showed low levels of mindfulness, suggesting that mindfulness may not work effectively in stressful situations [32]. Similarly, in our study, the high-stress group (M = 23.67) showed a lower level of mindfulness than that of the low-stress group (M = 25.73). Therefore, when the stress level is high, further mental health intervention strategies are needed in addition to mindfulness. In a study conducted in Korean undergraduate students, there was a partial moderating effect on the relationship between stress and depression, i.e., only one sub-concept of mindfulness (nonreactivity) had an interaction effect on the impact of interpersonal stress on depression, not a significant explanatory power change [13]. A study conducted in female undergraduate students in the United States reported that the mediating effect of mindfulness on their negative mental health state varies depending on the level of mindfulness [45]. As such, in order to examine the relationship between mindfulness and mental health outcomes more clearly, a more specific view of variables seems necessary. In this study, meaningful results were obtained by considering the degree of stress. Mindfulness was shown to play a role in reducing the intensity of the effects of stress on depression in the low-stress group. These findings imply that mindfulness is more useful in promoting mental health in daily life or preventing future mental health problems than it is in situations of high stress. Although there was no significant moderating effect of mindfulness in the high-stress group, mindfulness is still useful, as it has been shown to relieve stress and depression or act as a predictor of depression. In high-stress situations among university students, devising additional mental health strategies besides mindfulness will help to maximize the effectiveness of intervention. Therefore, it would be helpful to consider the stress levels of students to create more effective intervention strategies to deal with stress and students’ particular situations.

These study findings have significance in identifying the complex relationship between stress, mindfulness, and depression in a precise way, but there are limitations based on literature published in English and Korean. As a result of the mental health outcomes of university students, the moderators affecting them must be interpreted in the sociocultural context to which they belong, so this point should be considered when applying the results of this Korean study to another context. The convenience sampling method and the high proportion of female students (74.4%) among the participants also limit the representativeness of the sample and hinder generalization of the research findings. In future research, we suggest considering stress levels in the development of a mindfulness-based program to manage the stress and depression of university students.

## 5. Conclusions

We explored the moderating effects of mindfulness on the relationship between stress and depression. As a result of the moderated regression analysis, only the low-stress group showed a buffering effect of mindfulness on the impact of stress on depression. In the future, we suggest considering stress levels in the development of mindfulness-based intervention strategies to effectively manage the depression of university students.

## Figures and Tables

**Table 1:** Differences in stress, depression, and mindfulness by general characteristics (*N* = 738).

General Characteristics			Stress	Mindfulness	Depression
Variables	Categories	n (%)	M ± SD		
Sex	Male	189 (25.6)	27.02 ± 17.39	25.25 ± 5.35	15.08 ± 9.79
	Female	549 (74.4)	36.41 ± 17.88	24.54 ± 4.72	19.18 ± 10.12
	t (*p*)		−6.27 (<0.001)	1.73 (0.084)	−4.84 (<0.001)
Age	0–19 ^a^	85 (11.5)	29.89 ± 13.61	24.13 ± 4.67	16.84 ± 8.53
	20–22 ^b^	450 (61.0)	35.99 ± 18.95	24.62 ± 4.76	19.01 ± 10.21
	23+ ^c^	203 (27.5)	31.33 ± 17.65	25.20 ± 5.24	16.71 ± 10.60
	F (*p*), Scheffe		7.15 (0.001), a, c < b	1.68 (0.188)	4.38 (0.013), c < b
Year	Second ^a^	219 (29.7)	30.84 ± 16.02	24.49 ± 4.31	16.88 ± 9.00
	Third ^b^	315 (42.7)	36.63 ± 19.50	24.67 ± 5.17	18.84 ± 10.69
	Fourth ^c^	204 (27.6)	33.35 ± 17.85	25.05 ± 5.04	18.37 ± 10.52
	F (*p*), Scheffe		6.81 (0.001), a < b	0.71 (0.492)	2.48 (0.084)
Subjective	Bad ^a^	79 (10.7)	47.29 ± 18.45	24.62 ± 4.87	22.1 ± 11.25
economic	Moderate ^b^	347 (47.0)	36.34 ± 17.44	23.77 ± 4.63	19.31 ± 9.66
status	Good ^c^	312 (42.3)	28.38 ± 16.76	25.81 ± 4.97	15.81 ± 9.97
	F (*p*), Scheffe		42.35 (<0.001), a > b > c	14.95 (<0.001), b < c	17.17 (<0.001), a, b > c
Subjective	Bad ^a^	70 (9.5)	45.76 ± 18.78	23.27 ± 4.24	26.59 ± 11.66
health status	Moderate ^b^	303 (41.0)	37.91 ± 17.95	23.62 ± 4.44	21.01 ± 9.82
	Good ^c^	365 (49.5)	28.51 ± 16.37	25.92 ± 5.08	14.12 ± 8.23
	F (*p*), Scheffe		42.44 (<0.001), a > b > c	22.95 (<0.001), a, b < c	78.05 (<0.001), a > b > c

*M* = mean; SD = standard deviation; In the Age variable, “a” is the 0–19 category, “b” is the 20–22 category, and “c” is the 23+ category. In the Year variable, “a” is the Second category, “b” is the Third category, and “c” is the Fourth category. In the Subjective economic status variables, “a” is the Bad category, “b” is the Moderate category, and “c” is the Good category. In the Subjective health status variable, “a” is the Bad category, “b” is the Moderate category, and “c” is the Good category.

**Table 2:** Correlations among key variables (*N* = 738).

Variables	Stress	Mindfulness	Depression
	r (*p*)		
Stress	1		
Mindfulness	−0.284 (<0.001)	1	
Depression	0.625 (<0.001)	−0.431 (<0.001)	1

**Table 3:** Descriptive statistics for the low- and high-stress group (*N* = 738).

Variables		Low-Stress Group (*N* = 377)	High-Stress Group (*N* = 361)	Total Group (*N* = 738)
Stress	Range	3–31	32–90	3–90
	Median	21	46	31
	M ± SD (mean per item)	19.82 ± 7.65 (0.40 ± 0.15)	48.82 ± 13.67 (0.98 ± 0.27)	34.00 ± 18.21 (0.68 ± 0.36)
Mindfulness	Range	13–40	14–28	13–40
	Median	26	24	24
	M ± SD (mean per item)	25.73 ± 5.07 (2.57 ± 0.51)	23.67 ± 4.47 (2.37 ± 0.45)	24.72 ± 4.89 (2.47 ± 0.49)
Depression	Range	0–49	2–56	0–56
	Median	12	23	17
	M ± SD (mean per item)	12.99 ± 7.57 (0.65 ± 0.38)	23.50 ± 4.47 (1.17 ± 0.49)	18.13 ± 10.19 (0.91 ± 0.51)

M = mean; SD = standard deviation.

**Table 4:** Moderating effect of mindfulness between stress and depression in the low-stress group (*N* = 377).

Independent Variables	Model 1			Model 2			Model 3			Model 4		
	β	t	*p*	β	t	*p*	β	t	*p*	β	t	*p*
Sex	0.16	2.84	0.005	0.03	0.53	0.597	0.06	1.18	0.238	0.05	0.94	0.349
Age												
20–22	−0.01	−0.13	0.900	0.00	−0.00	0.998	0.00	−0.01	0.994	−0.01	−0.13	0.898
23+	−0.02	−0.22	0.826	−0.02	−0.31	0.755	−0.01	−0.14	0.888	−0.03	−0.44	0.663
Subjective economic status												
Bad	−0.01	−0.27	0.784	−0.04	−0.76	0.447	−0.04	−0.96	0.336	−0.06	−1.27	0.207
Moderate	0.07	1.29	0.196	0.01	0.19	0.846	−0.05	−1.00	0.317	−0.07	−1.38	0.168
Subjective health status												
Not satisfied	0.25	4.99	<0.001	0.20	4.21	<0.001	0.17	3.73	<0.001	0.16	3.60	<0.001
Moderate	0.19	3.65	<0.001	0.16	3.32	0.001	0.12	2.51	0.013	0.12	2.70	0.007
Stress (A)				0.40	8.31	<0.001	0.32	6.78	<0.001	0.33	6.98	<0.001
Mindfulness (B)							−0.33	−7.16	<0.001	−0.34	−7.45	<0.001
A × B										−0.11	−2.52	0.012
Model F (*p*)	8.41 (<0.001)			17.36 (<0.001)			23.24 (<0.001)			21.86 (<0.001)		
Adjusted R^2^	0.121			0.258			0.347			0.357		
R^2^ (ΔR^2^)	0.138			0.274 (0.136)			0.363 (0.089)			0.374 (0.011)		
ΔF (*p*)				69.09 (<0.001)			51.33 (<0.001)			6.36 (<0.012)		
